# A Spaceborne Synthetic Aperture Radar Partial Fixed-Point Imaging System Using a Field- Programmable Gate Array—Application-Specific Integrated Circuit Hybrid Heterogeneous Parallel Acceleration Technique

**DOI:** 10.3390/s17071493

**Published:** 2017-06-24

**Authors:** Chen Yang, Bingyi Li, Liang Chen, Chunpeng Wei, Yizhuang Xie, He Chen, Wenyue Yu

**Affiliations:** Beijing Key Laboratory of Embedded Real-time Information Processing Technology, Beijing Institute of Technology, Beijing 100081, China; yangchen@bit.edu.cn (C.Y.); libingyi_bit@bit.edu.cn (B.L.); chunpengwei_0302@163.com (C.W.); xyz551_bit@bit.edu.cn (Y.X.); chenhe@bit.edu.cn (H.C.); yuwenyue@racobit.com (W.Y)

**Keywords:** synthetic aperture radar (SAR), fixed-point, real-time processing, FPGA-ASIC hybrid, parallel acceleration

## Abstract

With the development of satellite load technology and very large scale integrated (VLSI) circuit technology, onboard real-time synthetic aperture radar (SAR) imaging systems have become a solution for allowing rapid response to disasters. A key goal of the onboard SAR imaging system design is to achieve high real-time processing performance with severe size, weight, and power consumption constraints. In this paper, we analyse the computational burden of the commonly used chirp scaling (CS) SAR imaging algorithm. To reduce the system hardware cost, we propose a partial fixed-point processing scheme. The fast Fourier transform (FFT), which is the most computation-sensitive operation in the CS algorithm, is processed with fixed-point, while other operations are processed with single precision floating-point. With the proposed fixed-point processing error propagation model, the fixed-point processing word length is determined. The fidelity and accuracy relative to conventional ground-based software processors is verified by evaluating both the point target imaging quality and the actual scene imaging quality. As a proof of concept, a field- programmable gate array—application-specific integrated circuit (FPGA-ASIC) hybrid heterogeneous parallel accelerating architecture is designed and realized. The customized fixed-point FFT is implemented using the 130 nm complementary metal oxide semiconductor (CMOS) technology as a co-processor of the Xilinx xc6vlx760t FPGA. A single processing board requires 12 s and consumes 21 W to focus a 50-km swath width, 5-m resolution stripmap SAR raw data with a granularity of 16,384 × 16,384.

## 1. Introduction

A spaceborne synthetic aperture radar (SAR) is a remote sensor that works in the microwave band. Its abilities to penetrate cloud cover and to collect data in the dark over large areas at high resolution make it unique compared to other imaging instruments [1]. As one of the important means of space-to-Earth observation, SAR plays an important role in disaster emergency response, environmental monitoring, resource exploration and geographic information access [2,3,4]. Recent publications have reviewed the applications of satellite remote sensing techniques for hazards manifested through solid Earth processes, including earthquakes, volcanos, floods, landslides, and coastal inundation [5,6,7].

In 1978, NASA’s SEASAT satellites demonstrated the ability of SAR to acquire high-resolution images. Since then, many countries have launched SAR satellites and carried out the research of spaceborne SAR processing. The Sentinel-1 mission, including both the S-1A (launched in 2014) and S-1B (launched in 2016) satellites, was specifically designed by the European Space Agency (ESA) to acquire data and information products for applications such as the observation of the marine environment, the surveillance of maritime transport zones, the mapping of land surfaces, and the offering of support during crisis situations [8]. The TanDEM-X/TerraSAR-X (TDX/TSX) constellation is a high-resolution interferometric SAR mission of the German Aerospace Center (DLR) intended to fulfil the requirements of a global homogeneous and high-resolution coverage of all land areas, thereby providing the vital information for a variety of applications [9]. ALOS-2 (launched in 2014) is the follow-up Japan Aerospace Exploration Agency (JAXA) L-band SAR satellite mission to ALOS. The overall objective is to provide data continuity for cartography, regional observation, disaster monitoring, and environmental monitoring [10]. The Korea Multi-Purpose Satellite-5 (KOMPSAT-5) was launched in 2013 by the Korea Aerospace Research Institute (KARI), and the RADARSAT Constellation Mission (RCM) is scheduled for 2018 by the Canadian Space Agency (CSA). The SAR data are expected to be used mainly for maritime surveillance/national security, disaster management and ecosystem monitoring [11,12].

Most of the abovementioned missions impose high demands on the real-time performance of SAR data processing. On-board processing is undoubtedly an efficient solution. It allows a higher precision SAR data (larger bit-width) to be processed, which leads to better image quality and enables an optional image compression. It improves the downlink bandwidth utilization and provides quick feedback to the radar controller [13]. With these processed data products, decision makers can quickly plan and respond. On-board processing also reduces the cost associated with ground data-processing operations.

As early as 2000, the MIT Lincoln Laboratory began a study of the implementation of real-time signal processors for SAR front-end signal processing [14]. The processors were designed, based on MIT’s own VLSI bit-level systolic array technology, to have high computational throughput and low power implementations. Langemeyer et al. of the University of Hannover (Germany), proposed a multi-DSP system for real-time SAR processing using the highly parallel digital signal processor (HiPAR-DSP) technique in 2003 [15]. Its small volume and low power consumption make it suitable for on-board usage in compact air- or spaceborne systems. The jet propulsion laboratory (JPL) has also worked to develop on-board processing. An experimental SAR processing system based on VLSI/SOC hardware was proposed [13]. A fault-tolerant FPGA (Xilinx Virtex-II Pro)-based architecture was proposed and tested using the SIR-C data [16,17]. The University of Florida developed a high-performance space computing framework based on a hardware/software interface in 2006 [18]. An FPGA serves as the co-processor/accelerator of the CPU. A near-real-time SAR processor (NRTP) was developed by the Indian Space Research Organization (IRSO) based on the Analog Devices TigerSHARC TS101S/TS201S DSP multiprocessor. On-board or on-ground quick-look real-time SAR signal processing is achievable for ISRO’s RISAT-1 [19]. With the rapid development of the storage capacity and computing capacity of the Commercial-Off-The-Shelf (COTS) FPGA, the state-of-art Xilinx Virtex-6 FPGA was adopted for the entire real-time SAR imaging system in 2013 [20]. In recent years, the graphics processing unit (GPU), with its large computing power, has also been used for real-time SAR processing [21].

As indicated by the development of on-board SAR real-time processing, building a high-performance SAR real-time processing platform for space deployment is hampered by the hostile environmental conditions and power constraints in space. The FPGA, ASIC, DSP, CPU and GPU are, to some extent, superior with respect to real-time processing. Although the GPU has a high processing power, its large power consumption makes it unsuitable for the harsh conditions of spaceborne on-board processing. The CPU and DSP take advantage of the design flexibility. However, they cannot provide enough FLOPS per watt, which leads to a bottleneck in their potential applications. Interconnection between multiple CPUs/DSPs will also bring additional and complex overheads to system design. Benefitting from its customized design, the ASIC can provide sufficient processing power. However, large-scale, complicated logic design requires a longer development period. The FPGA has made great progress in terms of on-chip storage resources, arithmetic logic resources and hardware-software co-design. It can adapt to a large throughput rate and strict real-time signal processing requirements.

In this paper we propose a hybrid heterogeneous parallel accelerating technique combining the advantages of both the FPGA and ASIC to realize an on-board real-time SAR processing system. As with many digital signal processing systems, such as OFDM and MIMO, we expect to adopt fixed-point processing to reduce the system scale and improve processing speed. Therefore, we conduct a computational burden analysis. A partial fixed-point processing scheme is proposed. The FFT, which is the main contributor to the processing burden, is implemented with fixed-point using the 130 nm CMOS technology. Complicated phase function generation calculations are implemented using the Xilinx FPGA. Based on the proposed fixed-point quantization error propagation model, the imaging quality is guaranteed. The system implementation results indicate that our design can potentially be used for spaceborne on-board real-time processing.

The rest of the paper is organized as follows: Section 2 reviews the CS algorithm and analyses the computational burden of the CS algorithm. Fixed-point quantization error propagation model is also proposed. In Section 3, we evaluate the imaging quality of the partial fixed-point system. Both the point target and actual scene are verified. In Section 4, the design methodology of the FPGA-ASIC hybrid heterogeneous parallel accelerating architecture is described. In Section 5, the corresponding hardware realization details and results are discussed. A comparison with related works is carried out to show the validity of our system. Section 6 concludes the paper.

## 2. Partial Fixed-Point Imaging Scheme

### 2.1. CS Algorithm Review

The CS algorithm is one of the most fundamental and popular algorithms for SAR data processing. As the kernel algorithm, the CS algorithm, with some pre- or post-steps, can process various modes, such as the stripmap, scan SAR, spotlight, sliding spotlight, Tops and Mosaic modes [22,23]. Compared with other algorithms, the CS algorithm retains reasonable efficiency [24]. The calculation of interpolation is replaced by phase multiplication in the processing of range cell migration correction (RCMC), which eliminates approximations and reduces the computational burden. Moreover, this algorithm can also solve the problem of the dependence of the secondary range compression (SRC) on the azimuth frequency because of the need for data processing in the two-dimensional frequency domain.

**Figure 1:**
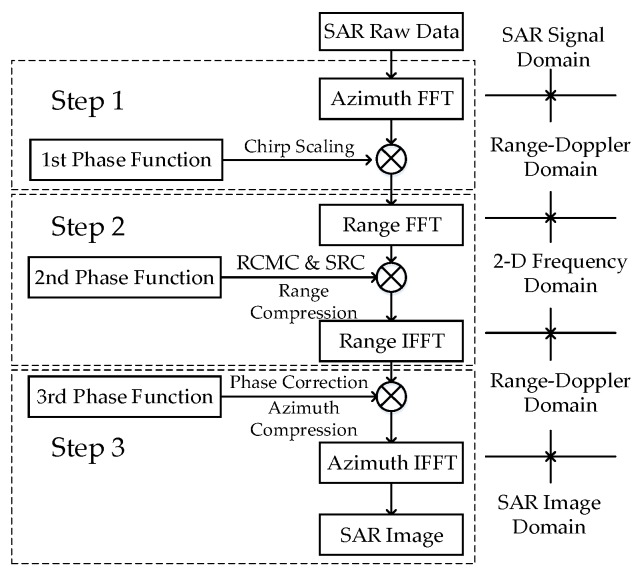
Flowchart of the CS algorithm.

The flowchart of the CS algorithm is illustrated in Figure 1. First, the SAR raw data are transferred to the Range-Doppler domain via a FFT in the azimuth direction. Second, the data are multiplied by the 1st phase function to achieve the chirp scaling, which makes all the range migration curves the same. The 1st phase function can be described as:

(1) $$\phi_{1}(\tau ,f_{\eta };r_{ref})=exp[-j\pi k(f_{\eta };r_{ref})c(f_{\eta }){\left(\tau -\frac{2}{c}r_{ref}(1+c(f_{\eta }))\right)}^{2}]$$

where τ is the range time, $f_{\eta }$ is the azimuth frequency, $r_{ref}$ is the reference distance, $k(f_{\eta };r_{ref})$ is the modulating frequency in the range direction, and $c(f_{\eta })$ is the curvature factor, expressed as:

(2) $$c(f_{\eta })=\frac{1}{\sqrt{1-{\left(\frac{\lambda f_{\eta }}{2v}\right)}^{2}}}-1$$

Third, the data are transferred to the two-dimensional frequency domain via a FFT in the range direction. Then, the data are multiplied by the 2nd phase function to complete the range compression, SRC and remaining RCMC. The 2nd phase function can be described as:

(3) $$\phi_{2}(f_{\tau },f_{\eta };r_{ref})=exp[-j\pi \frac{{f_{\tau }}^{2}}{k(f_{\eta };r_{ref})[1+c(f_{\eta })]}]exp[+j\frac{4\pi }{c}f_{\tau }r_{ref}c(f_{\eta })]$$

where $f_{\tau }$ is the range frequency.

Next, the data are transferred to the Range-Doppler domain via an inverse FFT in the range direction. The data can be multiplied by the 3rd phase function to complete the azimuth compression and phase correction. The 3rd phase function can be described as:

(4) $$\phi_{3}(\tau ,f_{\eta })=exp[-j\frac{2\pi }{\lambda }c\tau (1-\sqrt{1-{\left(\frac{\lambda f_{\eta }}{2v}\right)}^{2}})+j\frac{4\pi }{c^{2}}k(f_{\eta };r_{ref})(1+c(f_{\eta }))c(f_{\eta }){\left(r-r_{ref}\right)}^{2}]$$

Finally, the inverse FFT operation in the azimuth direction is executed to complete the CS algorithm. It can be seen that the algorithm only includes some FFT operations and multiplication operations. It is an effective and precise algorithm for stripmap SAR imaging.

### 2.2. Computational Burden Analysis

According to the flow chart shown in Figure 1, the CS algorithm is divided into three steps (Step 1–Step 3). The phase function generation mainly consists of scalar operations. Although the operations involved are complex, the computational burden is not large. From the perspective of the vector operations involved in the CS algorithm, the processing flow mainly consists of vector FFT/IFFT operations and vector complex multiplications. These operations can be decomposed into a combination of real additions and real multiplications. According to the classical Cooley-Tukey FFT algorithm [25], an *N*-point FFT/IFFT is decomposed into 3*N*log_2_*N* real additions and 2*N*log_2_*N* real multiplications. A complex multiplication is decomposed into two real additions and four real multiplications according to:

(5) (a+jb)×(c+jd)=(a×c−b×d)+j(a×d+b×c)

Assuming that the sample numbers of the range direction and azimuth direction are *N_R_* and *N_A_*, respectively, taking Step 1 as an example, one *N_A_*-point FFT as well as one complex multiplication is performed *N_R_* times. The total real addition computational burdens of the FFT and complex multiplication are 3*N_R_N_A_*log_2_*N_A_* and 2*N_R_N_A_*, respectively. The total real multiplication computational burdens of the FFT and complex multiplication are 2*N_R_N_A_*log_2_*N_A_* and 4*N_R_N_A_*, respectively. The computational burdens of the FFT and complex multiplication in different steps are shown in Table 1 and Table 2, respectively.

The percentage of FFT operations to the total computational burden is expressed as:

(6) $$P=\frac{10N_{R}N_{A}\log_{2}N_{A}+10N_{R}N_{A}\log_{2}N_{R}}{10N_{R}N_{A}\log_{2}N_{A}+10N_{R}N_{A}\log_{2}N_{R}+18N_{R}N_{A}}$$

For the convenience of hardware processing, the zero-padding method is usually adopted to expand the SAR raw data to an integer power of 2. Considering SAR raw data with a granularity of 16,384 × 16,384, the percentage *P* is over 90% according to (6). Therefore, the analysis result shows that the FFT accounts for the largest computational burden. We also test the time required for the 16,384-point FFT and the time required to generate the 16,384-point phase function, on both the standard CPU platform and the target FPGA platform. It takes the CPU (2× Intel Xeon E5-2697 (24 cores each) @ 2.7GHz) 4.9 ms and 0.2 ms to perform a 16,384-point FFT and to generate a phase function, respectively. It takes 16,500 clock cycles and about 400 clock cycles for the FPGA to perform a 16,384-point FFT and to generate a phase function, respectively. The experiment results also shows that FFT accounts for the largest computational burden. Fixed-point processing is faster and less resource intensive compared with floating-point processing. Adopting the fixed-point FFT is very important with respect to system hardware cost reduction and real-time performance improvement. On the other hand, phase function generation is computationally complex. It is necessary to ensure sufficient accuracy for the SAR image focus. We adopt the IEEE-754 standard single precision floating-point data format to generate the phase function.

### 2.3. Fixed-Point Processing Error Propagation Model

Due to the finite word length effect, fixed-point FFT processing introduces an additional quantization error. For the SAR imaging processing system, to meet the requirement of imaging accuracy, a proper processing word length should be determined. Blind simulation validation is inefficient and time consuming. Many works [26,27,28,29], which focus on word length optimization of DSP systems, tend to establish analytical models to analyse the mean value or variance of quantization noise. In our previous works [30], we also proposed the error propagation model to solve the finite word length effect of Range Doppler (RD) algorithm. Thus, we proposed an error propagation model to evaluate the quantization error power of the fixed-point processing CS algorithm. This will assist us in determining the word length of the FFT to a great extent.

**Figure 2:**
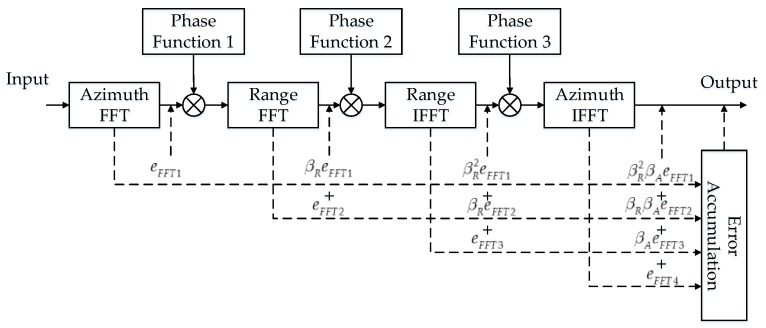
Proposed quantization error propagation model for CS algorithm.

The CS algorithm processing flow can be summarized as a cascade of multilevel FFT/IFFTs. Figure 2 shows the error propagation model. Taking the quantization propagation progress of the first azimuth FFT as an example, define $e_{FFT1}$ as the quantization error vector generated by the first azimuth direction FFT. β_R_ and β_A_ are the attenuation factors of the range direction FFT and the azimuth direction FFT, respectively. The quantization error is propagated through the latter range and azimuth FFT/IFFTs. The propagation progress of other FFT/IFFTs is similar.

The phase function generations and the complex multiplications are processed with single precision floating-point. The accuracy of the operations here ensures that no quantization errors are introduced. In addition, the complex multiplications do not attenuate the noise power. We prove this conclusion as follows. Define $e_{MUL1}$ as the quantization error vector after the first complex multiplication and $f_{1}$ as phase function 1. Then, $e_{MUL1}$ is expressed as follows:

(7) $$e_{MUL1}=e_{FFT1}\cdot f_{1}$$

Since the modulus of the phase function vector is 1, the power of $e_{MUL1}$ is derived as follows:

(8) $$P\_e_{MUL1}=E\left[{\left|e_{MUL1}\right|}^{2}\right]=E\left[e_{FFT1}f_{1}f_{1}^{H}{e_{FFT1}}^{H}\right]=E\left[e_{FFT1}{e_{FFT1}}^{H}\right]=E\left[{\left|e_{FFT1}\right|}^{2}\right]=P\_e_{FFT1}$$

Accordingly, the error power remains unchanged after a complex multiplication. Thus, we only take the FFT attenuation factors β_R_ and β_A_ into consideration.

According to [31], β_R_ and β_A_ can be assigned as 1/*N_R_* and 1/*N_A_*, where *N_R_* and *N_A_* are the sample numbers of the range direction and azimuth direction, respectively. Assuming that the quantization errors generated from different FFTs are mutually independent, the errors are finally accumulated at the output. The total quantization error output is derived as follows:

(9) $$e_{total}=\beta_{R}^{2}\beta_{A}e_{FFT1}+\beta_{R}\beta_{A}e_{FFT2}+\beta_{A}e_{FFT3}+e_{FFT4}$$

In our previous work [32], we revealed the quantization error assessment of the fixed-point FFT. The quantization error power of an *N*-point, *b*-bit fixed-point FFT is described as:

(10) $$P\_e_{FFT}(N,b)=2N\frac{2^{-2b}}{12}\log_{2}N$$

The total quantization error power is accordingly derived as follows:

(11) $$\begin{array}{cc} P\_e_{total} & =E\left[{\left|e_{total}\right|}^{2}\right]=\beta_{R}^{4}\beta_{A}^{2}E\left[{\left|e_{FFT1}\right|}^{2}\right]+\beta_{R}^{2}\beta_{A}^{2}E\left[{\left|e_{FFT2}\right|}^{2}\right]+\beta_{A}^{2}E\left[{\left|e_{FFT3}\right|}^{2}\right]+E\left[{\left|e_{FFT4}\right|}^{2}\right]\\ & =\frac{1}{N_{R}^{4}}\frac{1}{N_{A}^{2}}P\_e_{FFT}(N_{A},b)+\frac{1}{N_{R}^{2}}\frac{1}{N_{A}^{2}}P\_e_{FFT}(N_{R},b)+\frac{1}{N_{A}^{2}}P\_e_{FFT}(N_{R},b)+P\_e_{FFT}(N_{A},b)\\ & =\left(\frac{1}{N_{R}^{4}}\frac{1}{N_{A}}+N_{A}\right)\frac{2^{-2b}}{6}\log_{2}N_{A}+\left(\frac{1}{N_{R}}\frac{1}{N_{A}^{2}}+\frac{N_{R}}{N_{A}^{2}}\right)\frac{2^{-2b}}{6}\log_{2}N_{R} \end{array}$$

## 3. Fixed-Point Imaging Quality Evaluation

**Figure 3:**
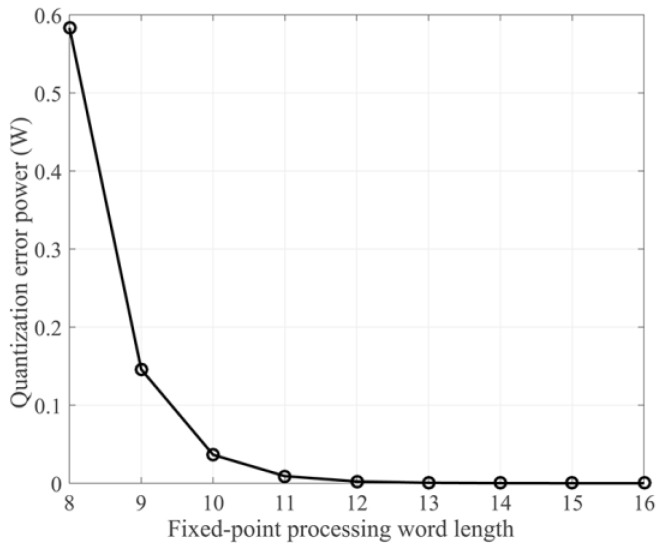
Quantization error power for different fixed-point processing word lengths.

Figure 3 shows the quantization error power under different processing word lengths according to (11). As the word length increases from 8 to 11, the quantization error power gradually decreases. As the word length increases from 12 to 16, the quantization error power remains essentially unchanged. Therefore, we can narrow the scope of the word length for verification. In the following subsections, we evaluated the imaging quality of both a point target and an actual scene for 12- to 16-bit word lengths.

### 3.1. Point Target Imaging Quality Evaluation

**Figure 4:**
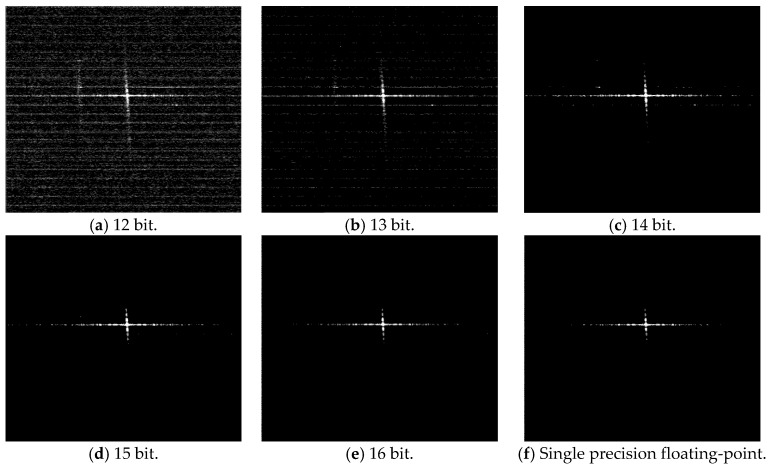
Point target imaging results for different FFT processing accuracies. (**a**–**e**) 12–16 bit fixed-point FFT; (**f**) single precision floating-point FFT.

Figure 4 shows the point target imaging results for different FFT processing accuracies. Figure 4a–e are the imaging results of the partial fixed-point processing based on SystemC, which is a bit-accurate hardware design verification language [33]. Figure 4f is the imaging result of a typical single precision floating-point processing based on MATLAB (or some other conventional ground-based software processors). We choose the floating-point imaging result as the accurate one for comparison.

For the point target, the peak sidelobe ratio (PSLR), integrated sidelobe ratio (ISLR) and spatial resolution (RES) are commonly adopted to evaluate the imaging quality [34]. Table 3 shows the imaging quality assessment comparison result. Theoretically, the squint angle of the side-looking SAR echo is zero. When generating the point target simulation data, there will be a slight error in the squint angle (less than 1 °). Thus, we set the squint angle to 1 ° when evaluating the imaging quality. Similar to the quantization error power curve, the imaging quality with a 15 or 16 bit word length is very close to that of single precision floating-point. The 15 or 16 bit partial fixed-point system seems to provide sufficient accuracy for the point target imaging.

### 3.2. Actual Scene Imaging Quality Evaluation

**Figure 5:**
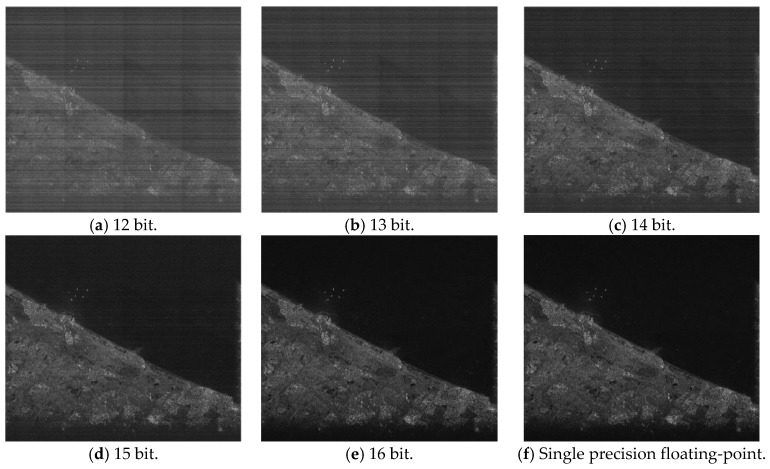
Actual scene imaging results (overall region) for different FFT processing accuracies. (**a**–**e**) 12- to 16-bit fixed-point FFT; (**f**) single precision floating-point FFT.

**Figure 6:**
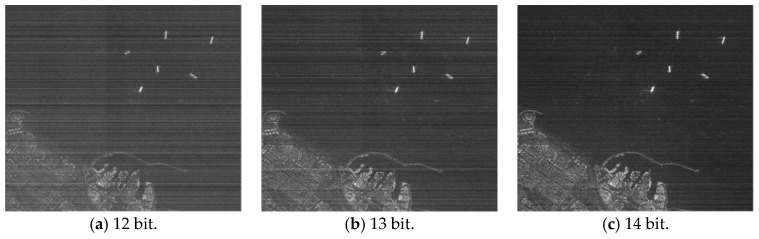

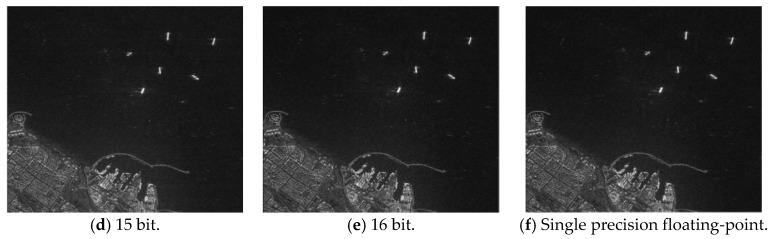
Actual scene imaging results (local region) for different FFT processing accuracies. (**a**–**e**) 12- to 16-bit fixed-point FFT; (**f**) single precision floating-point FFT.

Figure 5 shows the actual scene imaging results for different FFT processing accuracies. The raw data were obtained from the Chinese Gaofen-3 satellite’s 50-km-width, 5-m-resolution stripmap mode. Usually, the region of interest (ROI) extraction, target recognition and other SAR image applications are performed after the SAR imaging process. Thus, we also selected some local regions in the actual scene to evaluate. Figure 6 shows the imaging results.

For actual scenes, the mean squared error (MSE) is commonly adopted to evaluate the imaging quality. It is computed by averaging the squared intensity difference between fixed-point image pixels *x_i_* and floating-point image pixels *y_i_*. The peak signal-to-noise ratio (PSNR) is derived from the MSE [35]. The MSE and PSNR between two images are calculated as follows:

(12) $$MSE=\frac{1}{N}\sum_{i=1}^{N_{1}}{\left(x_{i}-y_{i}\right)}^{2}$$

(13) $$PSNR=10\log_{10}\left(\frac{L^{2}}{MSE}\right)$$

*N* is the total number of pixels in the image, and *L* is the maximum dynamic range. For 8-bit greyscale images, *L* = 255.

The MSE is easy to calculate and understand but cannot match the perceived visual quality very well. Therefore, based on the known characteristics of the human visual system (HVS), we also adopt structural similarity (SSIM) to evaluate the imaging quality [36]. The expression of SSIM is as follows:

(14) $$SSIM\left(x,y\right)=\frac{\left(2\mu_{x}\mu_{y}+C_{1}\right)\left(2\sigma_{xy}+C_{2}\right)}{\left(\mu_{x}^{2}+\mu_{y}^{2}+C_{1}\right)\left(\sigma_{x}^{2}+\sigma_{y}^{2}+C_{2}\right)}$$

$\sigma_{x}^{2}$ and $\sigma_{y}^{2}$ represent the variances of the fixed-point image and floating-point image, respectively. $\mu_{x}$ and $\mu_{y}$ represent the mean values of the fixed-point image and floating-point image, respectively. *C*_1_ and *C*_2_ are constant values. *SSIM* = 1 indicates that two images are nearly the same. Moreover, the radiometric resolution (γ) is also a very important indicator [37]. It reveals how fine a sensor can distinguish between objects with similar reflection properties. σ and μ represent the standard deviation and the mean value of the image, respectively. It is calculated as follows:

(15) $$\gamma =10\log_{10}\left(\frac{\sigma }{\mu }+1\right)$$

Table 4 and Table 5 show the imaging quality assessment comparison results of both the overall region and local region of the actual scene. The MSE as well as the PSNR is only evaluated for the overall region. Both the imaging results and the quality assessment indicate that the partial fixed-point system with a 16-bit fixed-point FFT processing word length can provide sufficient accuracy for the CS algorithm. According to the evaluation results for both the point target and actual scene, we choose to adopt the 16-bit fixed-point FFT in the actual system implementation.

SAR data intrinsically consist of amplitude and phase information. The above assessments quantify the impact of fixed-point processing from the amplitude information, while the accuracy of the phase information also needs to be validated. The SAR interferometry is based on the phase information in SAR complex products. In general, a pair of interferometric data acquired by different orbits is processed by the same CS algorithm to obtain two focused complex images of the actual scene. After the registration processing of the two complex images, the mean values of phase and phase standard deviation are estimated by the method proposed in [38]. Table 6 shows the phase information assessment comparison results of both the fixed-point and single precision floating-point processing of the actual scene. According to the phase information evaluation results, 16-bit fixed-point FFT is also appropriate for the actual system implementation.

## 4. FPGA-ASIC Hybrid Heterogeneous Parallel Accelerating Architecture

### 4.1. Hardware Design Methodology

The key principles followed in the system hardware design are as follows.

#### 4.1.1. Real-Time Operation

Requiring the processor to be able to operate without large power-hungry computers implies an efficient mapping methodology. The process of mapping the architecture and algorithm is to progress from a correct algorithm, described at a level of abstraction that is both implementation-independent and timing-independent, to a system description of time-dependent, specific allocation to processing resources and the sequencing of events within those resources. This mapping procedure is divided into static description and dynamic description. During static description, the procedure focuses on how to allocate each step or algorithm module to distinct processors with different characteristics in a hybrid heterogeneous system. A large amount of rapid but repetitive computations can be calculated by fixed-point ASIC co-processors. The FPGA contains a large number of user-defined floating-point intellectual property (IP) cores, which are more suitable for the less frequent but non-linear computations. Thus, the slower and often more irregular computations are assigned to the FPGA. In addition, a mass storage resource is required to store the large SAR raw data. Another task of the FPGA is to build a bridge between the storage and co-processors. During dynamic description, introducing time-area constraints helps to achieve a good balance between processor parallelism and processing latency. The procedure only examines the feasibility of algorithm parallel operational interconnection between processors during the runtime and does not yet look inside a processor for processor-specific features. 

#### 4.1.2. Accuracy and Flexibility

The partial fixed-point system framework is achieved based on our fixed-point error propagation model, as described above. Simultaneously, the bit-level accurate simulation method based on an advanced programming language (SystemC) provides a benchmark to gauge the performance of the processor. The simulation result of the software allows for verification of the segmentation of the algorithm, which provides a convenient condition for hardware modular design. The algorithm-based changes can be easily incorporated into the reconfigurable FPGA flexibility.

### 4.2. Static Mapping Strategy

**Figure 7:**
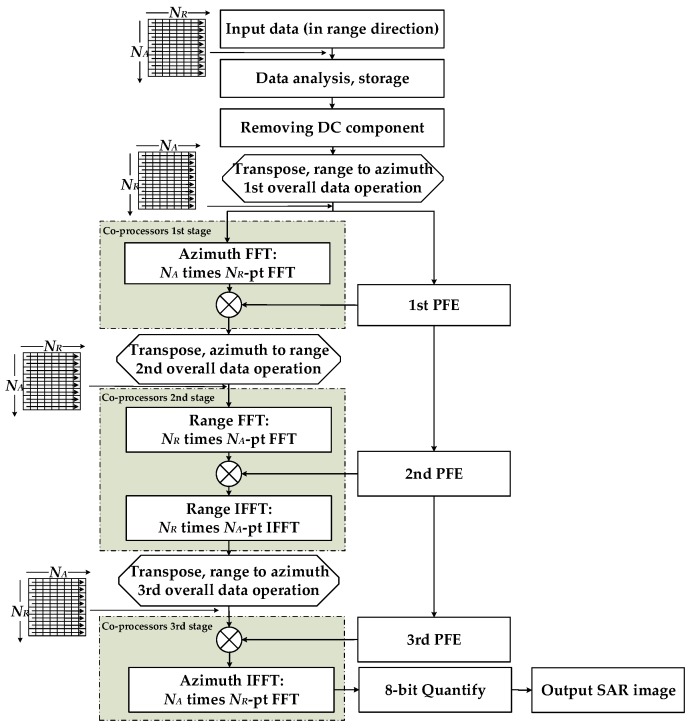
Processing chain for the CS SAR imaging algorithm.

The processing elements selected for this system design are the FPGA and fixed-point FFT co-processors. A Dual Data Rate (DDR) SDRAM is also introduced into the system; it acts as the external storage medium for the SAR raw data. In this paper, we only discuss SAR imaging processing after A/D conversion. The static mapping of CS image processing can be described by the end-to-end processing chain shown in Figure 7.

SAR raw data are first accessed in the FPGA. Then, the standard CS algorithm can be partitioned into two parallel branches after the preprocessing operations, i.e., the raw data analysis and storage and the direct current (DC) removal operation. One branch, denoted by the grey, dashed rectangular boxes, is processed by the co-processors. The other branch, denoted by the solid-line rectangular boxes, is processed by the FPGA. The FPGA and co-processors are controlled by independent instructions. Thus, they can work synchronously. For the FPGA part, since the three-fold phase function estimation (PFE) can be classified as a linear combination of transcendental operations, the development can be improved effectively through using the IP cores provided by specific development tools such as Xilinx ISE. For the co-processors part, the order of the FFT (IFFT) and multiplication units is flexible and can be configured according to the algorithm requirements. The quantify operation should be added after the last azimuth IFFT to achieve the greyscale images.

**Figure 8:**
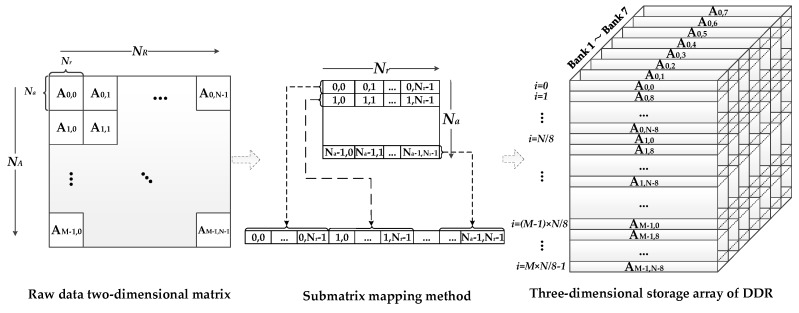
Submatrix three-dimensional mapping method.

The FPGA, as the storage medium, establishes a data access link between the co-processor and external DDR SDRAM. In Figure 7, the left branch shows that three overall data reading and writing operations occur before the three co-processor processing stages. In general, the SAR raw data are stored range-first. Thus, three matrix transpose operations are required during the total processing procedure. The DDR-based matrix transpose is achieved by using the submatrix three-dimensional mapping method [39]. In this way, the transpose operation can be performed simultaneously with data reading before the co-processors stage. The accessing of situ address data is the prerequisite of this method. The main procedure of this technology is shown in Figure 8, according to the following two steps:

1. Divide the *N_A_* × *N_R_* raw data matrix into *M* × *N* submatrixes of *N_a_* × *N_r_* order;
2. Map each submatrix into the three-dimensional storage array of the DDR. Each submatrix corresponds to a row of the DDR.

Considering that read and write accesses to the DDR are burst-oriented, *m* × *N* samples (*m* means the burst length) can be stored to or loaded from the DDR during *N* clock cycles. Therefore, in our design, the range or azimuth access mode can be described as follows.

#### 4.2.1. Range Access Mode

**Figure 9:**
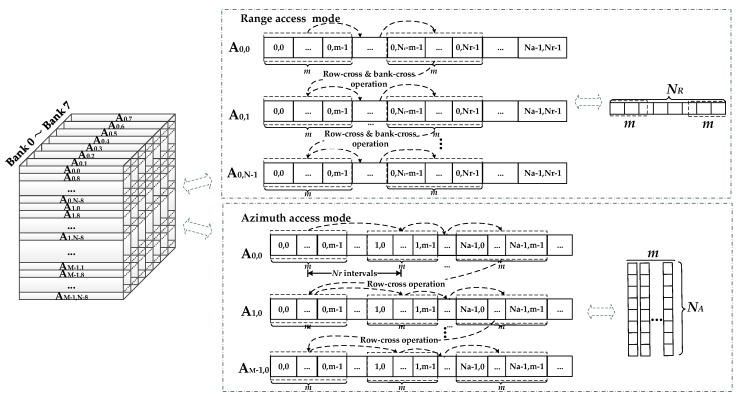
Range and azimuth access modes of the DDR SDRAM.

This mode corresponds to the overall data operation before the second co-processors stage. Take the first range reading operation line as an example. As shown in Figure 8, the data of this line are distributed into (0, 0) to (0, *N_r_*-1) of the A_0,0_ to A_0,N-1_ submatrixes. These submatrixes are mapped into the A_0,0_ to A_0,N-1_ rows of the DDR. Since each of the *m* data belongs to the same range line, the range line data can be accessed from each row of the DDR continuously. Therefore, the data are accessed in sequential order, with *m* data per clock cycle. *N* row-cross operations and *N*/8 bank-cross operations are performed when acquiring one range line of *N_R_* depth. This brings an additional data access time overhead. Thus, it takes more than *N_R_/m* clock cycles to obtain one range line of *N_R_* depth. The range direction reading operation described above is shown in the upper part of Figure 9.

#### 4.2.2. Azimuth Access Mode

This mode corresponds to the overall data operation before the first and third co-processors stages. Take the beginning *m* azimuth lines reading operations as an example. As shown in Figure 8, the data of these lines are distributed into the 0 to *m-*1 columns of the A_0,0_ to A_M-1,0_ submatrixes. These submatrixes are mapped into the A_0,0_ to A_M-1,0_ rows of the DDR. Different from the range access mode, each burst length consists of *m* data from *m* different azimuth lines. Thus, *m* azimuth lines data can be accessed at *N_r_* intervals, wit *m* data per clock cycle. *M* row-cross operations and no bank-cross operations are performed when acquiring *m* azimuth lines of *N_A_* depth. This also brings an additional data access time overhead. Thus, it takes more than *N_A_* clock cycles to simultaneously obtain *m* azimuth lines. The azimuth direction reading operation described above is shown in the lower part of Figure 9.

Figure 9 shows the reading operation of the two modes from left to right. The writing operation has just the opposite procedure compared to the reading operation. Apart from the influence of the line-cross and bank-cross operations of each access mode, the maximum bandwidth utilization of the DDR also depends on whether the internal FPGA cache memory can match these two completely different access forms. The relationship between the memory and parallel accelerating technique will be discussed in the following subsections.

### 4.3. Dynamic Mapping Strategy

Introducing the timing analysis of the critical path from the perspective of the hardware structure design is the main function of the dynamic description. This mapping strategy focuses on the following issues:

- Execution-time matching of parallel branch operations;
- Processing bandwidth allocations to multiple processors;
- Interaction between the different parts of the system (FPGA, co-processor and DDR).

This section mainly discusses the real-time performance of multiple co-processor parallel processing according to the above views. Theoretically, each range line or azimuth line FFT operation is independent of the others. This means that the more FFT processors there are, the better the system real-time performance achieved. However, the number of processors is determined based on the computational throughput requirements, which are restricted by the bandwidth of the FFT operations, PFE operations and DDR-FPGA communication. The relationship can be described as follows:

(16) $${\left[B_{ddr},kB_{m}\right]}_{\min}={\left[nB_{fft},B_{pfe}\right]}_{\min}$$

$B_{ddr}$ is the actual DDR effective bandwidth, which varies according to the access mode (range/azimuth). $B_{m}$ is the equivalent bandwidth of the FPGA memory, which is used to cache the data between the DDR and FFT co-processors. $B_{pfe}$ is the bandwidth of the phase function estimation operations. *k* represents the amount of FPGA internal cache memory required for a specific DDR access mode. Ideally, as described above, *k* should be 1 and *m* in the range access mode and the azimuth access mode, respectively. $B_{fft}$ represents the bandwidth of the FFT operations, and *n* is the parallelism of FFT co-processors. Actually, as mentioned before, the PFE is performed by a series of float-point IP cores in a pipelined steam. The simulation results show that the processing delay of the PFE operations is approximately dozens to hundreds of cycles. Therefore, the PFE is considered as a real-time operation, and $B_{pfe}$ can be ignored in (16). $B_{ddr}$, $B_{m}$ and $B_{fft}$ can be described as:

(17) $$B_{ddr}=\eta \rho B_{ddr\_peak}=2\eta \rho F_{ddr\_IO}W$$

(18) $$B_{m}=F_{m}W$$

(19) $$B_{fft}=F_{fft}W$$

$F_{ddr\_IO}$, $F_{m}$ and $F_{fft}$ represent the clock frequency of the DDR I/O, the FPGA internal memory and the FFT co-processor, respectively. *W* is the word length of the data processed per cycle. Since float-to-fixed and fixed-to-float operations should be done before and after the fixed-point FFT, respectively, a unified value of *W* can be adopted across the system as a general situation. $B_{ddr\_peak}$ is the peak bandwidth of the DDR, which can be represented by the product of double $F_{ddr\_IO}$ and *W*. η represents the actual DDR bandwidth loss ratio, which is determined based on the data access mode (range or azimuth) of the DDR. ρ is the utilization of burst length *m*. Consequently, the effectiveness of the parallel processing can be described as:

(20) $${\left[2\eta \rho F_{ddr\_IO}W,kF_{m}W\right]}_{\min}=nF_{fft}W$$

According to (20), the parallelism of co-processors is determined based on specific system features such as $F_{ddr\_IO}$, $F_{m}$, $F_{fft}$ and *W*.

## 5. Realization of the FPGA-ASIC Accelerating Platform

### 5.1. Implementation of the Customized Fixed-Point FFT Co-Processor

As discussed above, the FFT accounts for the largest computational burden. We take the standard FFT IP cores, which are provided by Xilinx FPGA, as an example to show the order of magnitude of computational efficiency improvement by exploiting fixed-point processing in FFT instead of floating-point. Table 7 shows the resource comparison between 16-bit 16,384-point fixed-point pipeline FFT processor and IEEE standard single precision floating-point pipeline FFT processor. The logic resource (registers, LUTs, and DSP48s) occupied by the floating-point FFT processor is more than twice that occupied by the fixed-point FFT processor. The memory resource (block RAMs/FIFOs) occupied by the floating-point FFT processor is three times as that occupied by the fixed-point FFT processor. The maximum working frequency of floating-point FFT processor is also lower than that of fixed-point FFT processor.

**Figure 10:**
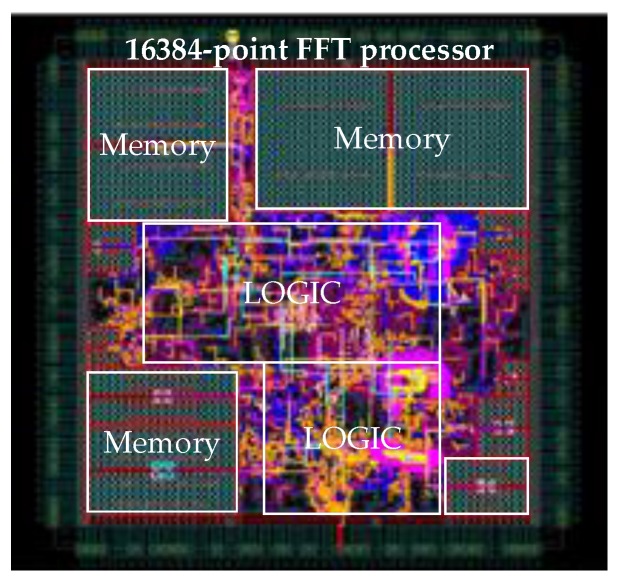
Layout of 16,384 point, 16-bit pipeline FFT processor.

In order to improve the flexibility of the system, reduce the system power consumption, and reduce the system requirements for the FPGA device selection, we adopt the ASIC implementation of the FFT processor. According to the fixed-point quantization error analysis, a 16,384 point, 16-bit FFT co-processor is implemented. It is a typical radix-2*^k^* single-path delay feedback (SDF) pipeline FFT processor based on our previous research [40]. The processing latency is approximately 16,500 clock cycles or 0.16 ms at a 100 MHz working frequency. The design is modelled in the VHDL language and synthesized using the Semiconductor Manufacturing International Corporation (SMIC, Shanghai, China) 0.13 μm standard cell library. Figure 10 shows the primary layout of the chip. Table 8 summarizes the main specifications of the chip. The total power consumption is only 84.9 mW @ 125 MHz. The chip turns out to be an energy-efficient tool for implementing the FFT, which is the most computationally intensive operation in the CS processing flow.

### 5.2. Parallel Processing Performance Analysis

To validate the effectiveness of the system design methodology, in this section, we establish a prototype using a single off-the-shelf Xilinx XC6VSX760T FPGA with fixed-point FFT co-processors to realize 16,384 × 16,384 stripmap SAR image processing. The raw data are represented in a single precision floating point complex form, which requires 64 bit (32 bit for the real part and for the imaginary part, respectively) for each sample data point. The DDR3 SDRAM with K4B4G0846Q-HYK0 SODIM is also introduced for the overall data storage. Table 9 shows the basic parameters of the system. $F_{fft}$ is derived from the ASIC design report. $F_{m}$ is limited by the maximum working frequency of the FPGA, which can be obtained from the synthesis report.

We adopt the ping/pong memory group as caches between the DDR and FFT co-processors. Considering the limitation of the FPGA resources, each group contains four SDRAMs with 0.125 MB of storage space. Each SDRAM is suitable for one 16 K × 64-bit data line.

The burst length *m* of the DDR3 SDRAM is 8. Thus, 8 × 64-bit data can be accessed in one cycle. For the range operation, the 8 × 64-bit data belongs to the same range line. It can be pipeline stored in the same DPRAM in eight clock cycles. Thus, the burst length utilization ρ is 1, and the amount of FPGA internal cache memory *k* is 1 in this case. According to the actual measurement, η is 0.9375. According to (20), the bandwidth of the FFT operations should match the bandwidth of the FPGA internal cache memory. Therefore, the co-processor parallelism *n* can be 2 to meet the requirements of range direction parallel processing.

For the azimuth operation, *k* is 4, limited by the number of DPRAMs of the ping/pong group. Thus, only the first 256 bit (4 × 64 bit) can be stored in parallel in the four DPRAMs (ping/pong group) in one cycle. Thus, ρ is 0.5 in this case. According to the actual measurement, η is 0.74. According to (20), the bandwidth of the FFT operations should match the bandwidth of the FPGA internal cache memory. Therefore, *n* can be 2 to meet the requirements of the azimuth direction parallel processing.

**Figure 11:**
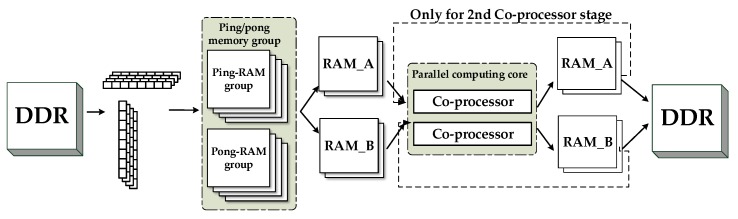
Unified flow chart of the parallel processing procedure.

Table 10 shows a summary of the parameters related to parallel processing. Combining the analysis results of both the range and azimuth operations, we adopt two FFT co-processors to perform the parallel acceleration for the SAR imaging system. The unified flow chart of the parallel processing procedure via a round-robin memory assignment is shown in Figure 11; the six steps of this procedure are as follows:

Step 1: Load four range or azimuth lines of data from the DDR to the ping (pong) SDRAM group.
Step 2: Divide the four DPRAMs of the ping (pong) group into pairs, named RAM_A and RAM_B, respectively. Select the data from RAM_A to send to the parallel FFT co-processors.
Step 3: Store the computation result to RAM_A after performing parallel processing using the FFT co-processors.
Step 4: Repeat steps 2 to 3 for the other two SDRAMs of RAM_B.
Step 5: Collect the data from both RAM_A and RAM_B and send them back to the DDR.
Step 6: Repeat steps 2 to 5 for the data from the pong (ping) group.

**Figure 12:**
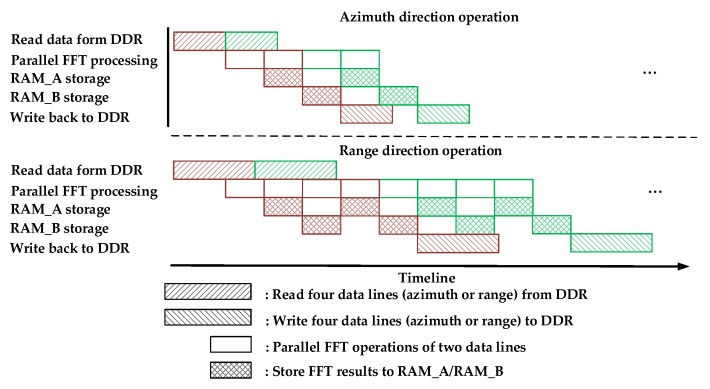
Parallel processing timeline of one round-robin assignment.

It is worth noting that there are both FFT and IFFT operations in the second co-processors stage, as shown in Figure 7. In this stage, Steps 2 to 3 should be executed once more before moving on to Step 4. The parallel processing timelines of one round-robin assignment for both the range direction operation and the azimuth direction operation are described in Figure 12. The red and green blocks represent the ping-memory-group-based operations and pong-memory-group-based operations, respectively.

### 5.3. Modular Design-Based System Architecture

**Figure 13:**
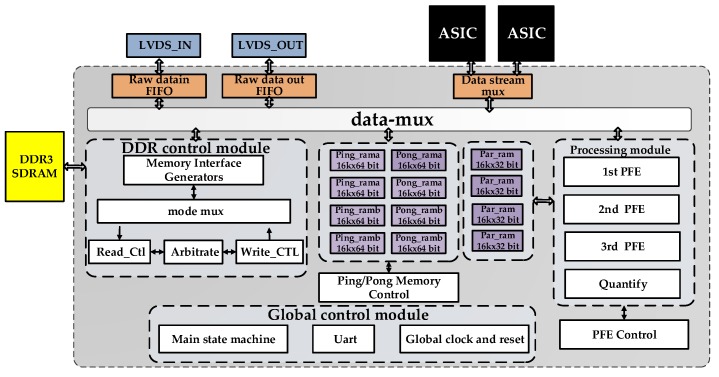
Block diagram of the SAR imaging system.

**Figure 14:**
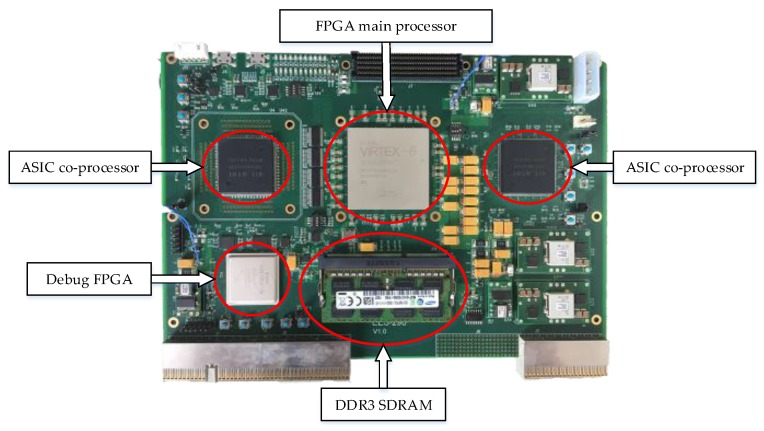
Photo of the hardware system.

The system block diagram and the photo of the hardware system are shown in Figure 13 and Figure 14, respectively; it is mainly composed of one FPGA, two ASICs as co-processors and one DDR3 SDRAM. The FPGA design follows the modular design principle. A Universal Asynchronous Receiver/Transmitter (Uart) is used to receive the external control commands from the host computer for single-step debugging. The accuracy of each step of the CS algorithm can be verified in this way. The other main function modules of the FPGA include the following:

- Customized processing modules: PFE operations module and quantify module.
- DDR control module: This module manages the data access mode between the DDR and main memory module. The memory interface generators (MIG) IP core is adopted to communicate with the DDR.
- Global control module: This module contains the main state machine, which is responsible for the phase transition, global clock and reset. Global information is propagated to all other modules in the form of broadcasts.
- Main memory module: The main memory module comprises the ping/pong memory group and four 16K × 32-bit dual port RAMs (Par_ram) and stores the intermediate parameter during the PFE stages.

The data interconnection between each module is realized by the 64-bit customized bus, which is called the data-mux in this design. We adopt the ordinary handshake agreement since each module has an independent control part to analyse the main state information. In practical image processing, SAR raw data are sent to the DDR3 SDRAM through the LVDS. By managing and combining different modules, the CS imaging algorithm is completed. The 8 bit greyscale image result after quantification can finally be outputted by the LVDS. The FPGA resource occupation is shown in Table 11.

**Figure 15:**
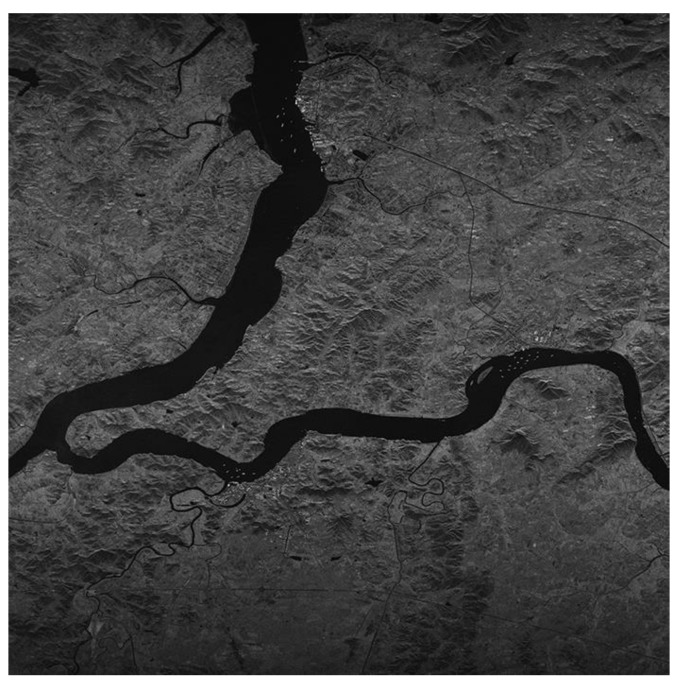
Imaging result of the system hardware.

According to the above timing analysis, it takes 12.1 s for the system hardware to process SAR raw data with 16,384 × 16,384 data granularity. Twenty-one watts are required for the whole system to work. Figure 15 shows the final imaging result of the proposed partial fixed-point system hardware. The image quality is reliable, which is consistent with the above SystemC-based software simulation results.

The pulse repetition frequency (prf) of Gaofen-3 satellite’s 50-km-width, 5-m-resolution stripmap mode is about 2000. Thus, it takes about 8 s to acquire the SAR raw data. Admittedly, the proposed single-board FPGA-ASIC processing system (12.1 s processing latency) just realizes a near real-time processing performance. However, through the construction of a multi-board parallel processing system, it is feasible to achieve a true real-time processing system. For a simple example, with the parallel processing of two boards, the processing latency is easily decreased to about 6 s, which meets the requirement of the data acquisition time.

### 5.4. Comparison of the Proposed System with Other Schemes

Table 12 shows a comparison between the proposed hybrid heterogeneous parallel system and some previous works. Compared with [2,19,41], taking into account the data granularity processed, the proposed system undoubtedly shows advantages in both processing time and power consumption. Ref. [42] describes a SAR image focuser application exploiting General-purpose Computing on Graphics Processing Units (GPGPU). The OpenCL framework allows the application to exploit to GPUs and CPUs. It takes 8.5 s to focus an ENVISAT ASAR’s stripmap SAR raw data. Taking into account the data granularity (30,000 × 6000), the processing time is comparable to ours. However, at the current level of technology, the GPU is more suitable for ground-based accelerators than onboard processors. FPGA has an inherent advantage in terms of FLOPS per watt. Although [21] only takes 2.8 s to process SAR raw data with 32,768 × 32,768 data granularity, the large power consumption of the GPU is unacceptable with respect to the harsh spaceborne onboard real-time processing requirements. In [43], the authors present a spaceborne SAR on-board processing simulator using the low-power mobile GPU. The low power consumption seems appealing and competitive for a SAR onboard processing task. However, according to their experiments on 1024 × 1024, 2048 × 2048 and 4096 × 4096 SAR imaging processing, the time utilization increases linearly due to the raw data size. Thus, it is reasonable to assume that the time utilization of a 16,384 × 16,384 SAR imaging processing is about 205 s. The processing time is unacceptable. In [44], the authors present a novel architecture for real-time SAR signal processing. Real time processing is achieved by using the Signum Coded algorathm in which raw data and reference function are coded with a single bit. Using this method can truly reduce the scale of the hardware implementation, including storage and computing resources. However, the method also has some limitations. If the signal/noise ratio (SNR) of the signal to be processed is high, false targets and high side lobe are generated after the imaging processing, and false targets become more obvious as the SNR increases. The image quality depends on the signal SNR, which to some extent affects the versatility of this method. In summary, the proposed design achieves a better tradeoff between real-time performance and power consumption.

## 6. Conclusions

In this paper, to complete the on-board real-time SAR imaging processing procedure in harsh space conditions, a partial fixed-point imaging system using the FPGA-ASIC hybrid heterogeneous parallel accelerating technique is proposed. With a proposed fixed-point processing error propagation model, the fixed-point processing word length is determined. The imaging fidelity is verified via both a point target and an actual scene imaging quality evaluation. Through a reasonable algorithm-to-system mapping, one Xilinx FPGA and two ASICs work together in parallel. The efficient architecture achieves high real-time performance with low power consumption. A single processing board requires 12 s and consumes 21 W to focus 50-km width, 5-m resolution stripmap SAR raw data with a granularity of 16,384 × 16,384. Admittedly, the proposed design is a prototype verification of the spaceborne on-board real-time processor. Both the system scale and the power consumption meet the harsh constraints of on-orbit processing. With the development of anti-radiation reinforcement technology and system fault-tolerant technology, the proposed framework is undoubtedly expandable and feasible for potential spaceborne real-time SAR imaging processing.

## Figures and Tables

**Table 1:** Computational burden of FFT/IFFTs in different steps.

Step No.	Real Additions	Real Multiplications	Total
Step 1	3*N_R_N_A_*log_2_*N_A_*	2*N_R_N_A_*log_2_*N_A_*	5*N_R_N_A_*log_2_*N_A_*
Step 2	6*N_R_N_A_*log_2_*N_R_*	4*N_R_N_A_*log_2_*N_R_*	10*N_R_N_A_*log_2_*N_R_*
Step 3	3*N_R_N_A_*log_2_*N_A_*	2*N_R_N_A_*log_2_*N_A_*	5*N_R_N_A_*log_2_*N_A_*
Total	6*N_R_N_A_*log_2_*N_A_* + 6*N_R_N_A_*log_2_*N_R_*	4*N_R_N_A_*log_2_*N_A_* + 4*N_R_N_A_*log_2_*N_R_*	10*N_R_N_A_*log_2_*N_A_* + 10*N_R_N_A_*log_2_*N_R_*

**Table 2:** Computational burden of complex multiplications in different steps.

Step No.	Real Additions	Real Multiplications	Total
Step 1	2*N_R_N_A_*	4*N_R_N_A_*	6*N_R_N_A_*
Step 2	2*N_R_N_A_*	4*N_R_N_A_*	6*N_R_N_A_*
Step 3	2*N_R_N_A_*	4*N_R_N_A_*	6*N_R_N_A_*
Total	6*N_R_N_A_*	12*N_R_N_A_*	18*N_R_N_A_*

**Table 3:** Point target imaging quality assessment for different FFT processing accuracies.

Word Length (bit)	Azimuth Direction			Range Direction		
	PSLR (dB)	ISLR (dB)	RES (m)	PSLR (dB)	ISLR (dB)	RES (m)
12	−5.72	−3.05	5.38	−5.91	−3.18	3.69
13	−8.17	−6.66	5.08	−8.75	−6.21	3.11
14	−11.87	−8.23	4.82	−11.56	−9.11	2.84
15	−12.56	−9.24	4.82	−12.88	−9.41	2.84
16	−12.82	−9.51	4.78	−13.26	−9.91	2.62
floating point	−12.89	−9.62	4.76	−13.29	−9.94	2.60

**Table 4:** Actual scene (overall region) imaging quality assessment for different FFT processing accuracies.

Word Length (Bit)	MSE	PSNR (dB)	SSIM	γ (dB)
12	2777.9	13.7	0.24	4.13
13	977.7	18.2	0.62	4.23
14	397.0	22.1	0.72	4.66
15	185.2	25.5	0.85	4.85
16	90.1	28.6	0.97	4.92
single precision floating point	0	∞	1	4.98

**Table 5:** Actual scene (local region) imaging quality assessment for different FFT processing accuracies.

Word Length (Bit)	SSIM	γ (dB)
12	0.24	0.36
13	0.45	0.61
14	0.56	1.01
15	0.77	1.28
16	0.93	1.33
single precision floating point	1	1.37

**Table 6:** Actual scene (overall region) phase information assessment for different FFT processing accuracies.

Word Length (Bit)	Phase Mean (°)	Phase Standard Deviation (°)
12	0.00918	3.3052
13	0.00581	3.3038
14	0.00401	3.3033
15	0.00270	3.3029
16	0.00254	3.3027
single precision floating point	0.00243	3.3027

**Table 7:** Resource comparison between 16,384-point fixed-point FFT processor and single precision floating-point FFT processor.

Parameters	16-bit Fixed-Point FFT Processor	Single Precision Floating-Point FFT Processor
Number of slice registers	3823	8247
Number of LUTs	3190	6864
Number of block RAMs/FIFOs	20	61
Number of DSP48s	24	60
Maximum working frequency	300 MHz	200 MHz

**Table 8:** Specifications of the FFT co-processor chip.

Parameters	Value	
Technology	130 nm CMOS	
Max Working Frequency	125 MHz	
Core Area	2.255 × 2.254 mm^2^	
IO supply voltage	3.3 V	
Internal voltage	1.2 V	
Pin Count	256	
Package	LQFP256	
Power with IO pads @ 125 MHz	I/O pads	29.9 mW
	Memory	37.1 mW
	Logic	17.9 mW
	Total	84.9 mW

**Table 9:** Basic parameters of the system implementation.

Parameter	Value
FPGA Main Frequency	100 MHz (Maximum 215 MHz)
*N_A_*	16,384
*N_R_*	16,384
*N_a_*	32
*N_r_*	32
*m*	8
$F_{ddr\_IO}$	400 MHz
$F_{m}$	200 MHz
$F_{fft}$	100 MHz
*W*	64 bit

**Table 10:** Summary of the parameters related to parallel processing.

Parameter	Range Direction Operation	Azimuth Direction Operation
η	0.9375	0.74
ρ	1	0.5
*k*	1	4
$B_{ddr\_peak}$	6.4 GB/s	6.4 GB/s
$B_{ddr}$	6 GB/s	2.37 GB/s
$B_{m}$	1.6 GB/s	1.6 GB/s
$B_{fft}$	0.8 GB/s	0.8 GB/s
*n*	2	2

**Table 11:** FPGA resource occupation (Xilinx xc6vlx760t).

Parameter	Value
Number of slice registers	98,219 (10%)
Number of LUTs	86,152 (18%)
Number of block RAMs/FIFOs	406 (56%)
Number of DSP48s	234 (27%)

**Table 12:** Comparison with previous works.

Works	Year	Schemes	Data Granularity	Working Frequency	Power Consumption	Processing Time
Proposed	2017	FPGA + ASIC	16,384 × 16,384	100 MHZ	21 W	12.1 s
[42]	2017	GPGPU	30,000 × 6000	-	-	8.5 s
[2]	2016	FPGA+ Microprocessor	6472 × 3328	-	68 W	8 s
[21]	2016	CPU + GPU	32,768 × 32,768	-	>330 W	2.8 s
[43]	2016	GPU	2048 × 2048	-	5 W	3.2 s
[41]	2012	CPU + ASIC	1024 × 1024	100 MHz	10 W	-
[19]	2008	Multi-DSP	4096 × 4096	100 MHz	35 W	13 s
[44]	1998	ASIC	1020 × 200	10 MHz	2 W	-
